# The Skeleton in the Closet: Faults and Strengths of Public Versus Private Genetic Biobanks

**DOI:** 10.3390/biom10091273

**Published:** 2020-09-03

**Authors:** Pamela Tozzo, Luciana Caenazzo

**Affiliations:** Department of Molecular Medicine, Laboratory of Forensic Genetics, University of Padova, 35121 Padova, Italy; luciana.caenazzo@unipd.it

**Keywords:** public biobank, private biobank, public trust, DTC genetic testing, genomic data sharing

## Abstract

Direct-to-consumer (DTC) genetic testing has been a major ethical controversy related to clinical utility, the availability of pre- and post-genetic counseling, privacy concerns, and the risk of discrimination and stigmatization. The development of direct-to-consumer genetic testing cannot leave aside some considerations on how the samples are managed once the analyses have been completed and the customer has received a response. The possibility that these samples are maintained by the structure for future research uses, explains the definition, which has been proposed in the literature, of these structures such as private genetic biobanks. The most relevant aspects that may impact ethical aspects, allowing a comparison between the public and private dimensions of genetic biobanks, are mainly transparency and participant/donor trust. The article aims to analyze the main line of ethical debate related to the mentioned practices and to explore whether market-based and consumer rights regarding DTC genetic testing can be counterbalanced by healthcare system developments based on policies that encourage the donation of samples in the context of public biobanks. A platform for dialogue, both technical–scientific and ethical, is indispensable between the public sector, the private sector and citizens to truly maximize both transparency and public trust in both contexts.

## 1. Introduction

The value of genetic information as well as policies dealing with access to genetic testing and/or findings resulting from these tests has been one of the most intriguing ethical issues recently discussed both in the scientific literature as well as in the media.

The sharing of a large amount of clinical and genetic data constitutes the basis of research, both in the genetic and clinical fields, for the improvement of the currently available biomedical knowledge. However, this approach requires not only the sharing of forms of technical governance, but also the sharing of regulatory and ethical principles that allow the management of this large amount of data while protecting citizens’ rights at the same time.

Direct-to-consumer (DTC) genetic testing are genetic tests commissioned directly, and the results of which are sent directly to the client, that is, without the intermediation of a healthcare professional. This type of service involves the analysis of more or less long sequences of genome, implying the derivation of a significant amount of data related to the sample that has been sent by the client. Most of the private facilities offering this service destroy biological samples after performing the required analyses, but some retain these samples for future use. The development of DTC genetic testing cannot leave aside some considerations on how the samples are managed once the analyses have been completed and the customer has received a response. The possibility that these samples and related data are maintained by the structure for future genomic and genetic research uses makes these structures data repositories. These data can be accessed by different entities for research and, for this reason, we can consider this as a sort of, also if mostly private, biobank [1,2]. It is clear that they do not fall into the traditional definition of the biobank, but the fact that they keep data and samples beyond the original objectives and that these samples can be exchanged /shared /sold makes them as forms, albeit atypical, of biobanks. DTC genetic testing companies create in this way their own biobank infrastructure in which customers’ genetic data and related phenotypic information are stored and used as in traditional biobanks of universities and hospitals. In this sense, biobank participants are derived from the customer base of the DTC genetic testing company [3].

Biobanks are “repositories which assemble, store, and manage collections of human specimens and related data. While the collection of samples and data for research purposes has a long history in the educational and medical systems, their recent increase in number, size, and importance has focused attention on the changing nature of biomedical research and relationships among investigators, research participants, and the organizations that fund and manage these entities” [4]. Biobanks, therefore, can be considered scientific infrastructure, as they are established according to the logic and practices of scientific research and respond to scientific projects and purposes.

Modern biobanks should be looked at as important structural platforms for the sharing of samples and data, providing a general benefit to scientific progress in regard to the sharing of objectives and samples for research. Therefore, it has been suggested that the characteristics, which should be respected when a biobank is designed and managed, are those of research services providers in order to reach a common governance platform. Interventions in terms of regulations and ethics on the issue of biobanking often feed the widespread impression that research is a potential threat to citizens, and that the role of regulations and ethics is mainly to protect citizens from this potential threat. However, respectful research is above all a resource for people; in this sense, regulations and ethics can play a role not so much in science as controllers, but as allies.

Among the reasons why people buy DTC genetic testing, there are two that are of greater interest for this discussion outline: to find out about their ancestry, and to obtain more information about their genetic risks and advice on disease prevention strategies. Despite numerous criticisms claiming that DTC genetic testing provides context-unrelated data that do not sufficiently inform the consumer, who is also exposed to privacy risks, DTC genetic testing has been developing into a booming industry of services. Furthermore, another kind of ethical concern has emerged in relation to DTC genetic testing offering the promise of the return of so-called “actionable” health-related findings as a benefit, which is a guarantee to those who turn to this type of biobank. For these reasons, DTC genetic testing has been a major controversy related to clinical utility, the availability of pre- and post-genetic counseling, privacy concerns, and the risk of discrimination and stigmatization. Concern has also be heightened for particular areas of genomic and other health-related information, which include information relating to ascertained or presumed genetic predispositions, such as information about potential genomic contributors to drug or substance abuse, propensity for criminal behavior, and intelligence or impulsivity, as well as particular kinds of research uses, such as studies that stratify participants by social or ancestry groups.

The ethical aspects relating to the relationship between individual companies and individual customers therefore catalyzed attention in recent years. However, there is another aspect of this DTC genetic testing that deserves to be explored, and it is related to the possibility of considering these structures to be private biobanks, with different assumptions but similar research purposes compared to public genetic biobanks. However, if the research purposes are the same, and only prerequisites are different, is it possible to compare these two types of reality and therefore understand why these private genetic biobanks seem to enjoy greater success than public ones in terms of visibility and public engagement considering that customers have to pay to obtain health-related and genetic information [5]. The failure of public biobanks related to clinical-data-sharing initiatives has been partly attributed to a failure to obtain public trust. A recent review of population attitudes towards biobanks has reported that the donors may be discouraged by, among other things, inadequate knowledge of biobanking and concerns over the use of the sample in line with donors’ values. In addition, many respondents were afraid of stigmatization and discrimination as well as the commercial use of their samples [6]. Lack of trust in this initiative seems to be mainly related to public concerns about transparency, confidentiality and privacy. The interest in comparing these two types of biobanks also derives from the fact that, recently, often effective models of collaboration between the public and private sectors have developed, so different studies on how such collaborations can take off have become interesting from the point of view of ethical, legal and societal implications (ELSI) [7].

This descriptive commentary aims to analyze the main line of ethical debate related to the mentioned practices and to explore whether market-based and consumer rights regarding DTC can be counterbalanced by healthcare system developments based on policies that encourage the donation of samples in the context of public biobanks.

In this descriptive analysis, the authors compare public and private biobanks with respect to their success with the public on the basis of two general aspects that have ethical implications: transparency and trust of the participants. The analysis of these aspects should take into account that, whereas in public biobanks donors become participants when engaged in their governance, in private biobanks, it is the customers who become donors when their samples, sent for diagnostic reasons (often paying a fee, and not donated), become samples used for research. Although this manuscript is focused on genetic testing, it should be clear that this discussion may hold true for all kinds of molecular testing (i.e., genetic, epigenetic and protein-based).

## 2. Biobanks Are Still Debating

As stated in Time Magazine in March 2009, biobanks were one of the ten ideas that changed the world [8]. Biobanking aims to collect biological samples and extract data from them in order to carry out research projects in the biomedical field. Indeed, pharmacogenomics and individual care plans based on each person’s DNA are becoming more common and cheaper. Some countries have created their own biobanks where they can store samples according to their laws, which can differ a great deal from one country to another [9].

Over the past decade or so, private biobanking has gained considerable traction with the emergence of powerful actors, such as 23andMe, Ancestry and MyHeritage. These players have gained a very large portion of demand in their respective markets, dwarfing their public counterparts in a very short amount of time. This trend can be seen worldwide, not only in countries like the USA or the UK where the biggest private sector players in the world originated, but also in countries like Italy with the notable biobank of Dante Labs.

These companies seem to be more dynamic and endearing than their public counterparts, they usually offer a service that is very specific and sought after in the era of tailored DTC services, their delivery is advertised as fast and curated as a health-related service is supposed be, and they do so at an ever-decreasing price. As of today, the 23andMe website offers an array of tests that range from USD 79 for their Ancestry + Traits service, USD 149 for their Health + Ancestry service, and up to USD 499 for their VIP Health + Ancestry service, with only the latter providing one year “premium customer support”. Another higher end service is that of Dante Labs, based in Italy, who offers more expensive but more complete genome sequencing, starting from USD 323.54 for their Premium Test, and from USD 918.69 for their Super Premium Test.

A big part of their appeal resides in the apparently easy access to the results, with a brief explanation of the content of the genomic sequencing results, which would otherwise be unintelligible for the average consumer without a medicine degree. However, this is also an issue and not only a positive feature of their offering, because without specialist support, customers will not be able to correctly understand the meaning of his or her complex results. For example, Dante Labs reports, as with most of these services, pharmacogenetics results and will tell customers if they are in a high, medium or low risk of a sensitivity range for a certain substance (ethanol, aspirin, cisplatin or nicotine just to name a few). Most customers’ attention is focused on the high-, medium- or low-risk sensitivity tag, therefore putting in the background the fact that the results just report that a variant related to a possible reaction has been found, that is, the subject may be placed under a high-risk tag while being not sensitive to the substance.

Then, if one cares to expand upon a high-risk tag of sensitivity, one will also obtain additional information such as the genotype, the gene, the position and the marker of the related result, and why a certain result is present. The amount of information given to the customer is huge, and the effort required reading through and, most importantly, understanding what he or she reads is so substantial that the issue of lack of counselling is immediately evident. Counselling is usually offered only for the higher-end testing, or with payment of a supplemental fee and only for a limited amount of time. In the case of 23andme, the information relating to the meaning of the tests is provided in a dedicated section of the website, in which prudence is predominant: the suggestion to contact a geneticist consultant is clearly highlighted and a link is provided that directs the user to a page managed by the National Society of Genetic Counselors.

Dante Labs is a service of undoubtable quality, and on their web pages, as with all of the major private biobank services, they provide information about privacy issues, consent, use of the data and of the biological samples they collect, and they always provide customer service interfaces. This is all in stark contrast with the public biobanking services that one can access through the web, where the websites are harder to navigate by design, less capturing of one’s attention and often not complete with the information a donor would need both to decide to donate and to understand what such a choice would entail. Take the CRB (center for biological resources) of the Italian San Raffaele Hospital, or the Italian Biobank of the Spallanzani Hospital as two representative examples of this apparently superficial but fundamental issue. If further examples are required of the Italian public biobanking situation, one can visit the Biobanking and BioMolecular Resources Research Infrastructure of Italy (https://www.bbmri.it/) website that lists a great many public biobanks. However the two cited examples are a good proxy for publicly funded health institutions. At an international level, in the case of 23andme, the way in which customers are asked to allow the use of their samples for research and to provide a series of personal information to be used for research is emblematic: all terminology is centered on “power“ to the customer; the customer has the power to decide and has a heavy impact on the future of research and medicine, meaning that in a revolutionary and fast way you can make a difference in the discovery of new diseases and in the treatment of those most well-known.

## 3. Discussion

The fundamental differences that arise between these two sides of the same coin are what might have been the cause of the underperforming of public-sector biobanking efforts over the past few years, as their private-sector counterparts are flourishing and expanding faster than ever. These differences are image related, as public biobanks come off as less transparent, harder to reach, slower, and provide a lower payoff for the donor—if not a negative one, as most of them do not even provide the results of the conducted testing to the respective donor. Furthermore, public biobanks do not incentivize the donor enough, often not moving beyond the implicit claim that through individual donors’ action the public will eventually be better off.

The differences between public and private are also communication related. That is, the donor will only decide to donate out of altruism and opportunity, with opportunity usually meaning that a doctor confronted with a patient suffering from a specific condition will let them know that there is a biobank dedicated to the harvesting of samples used to further research about such a condition, and ask them if they care to donate their sample. Otherwise, public biobanks seldom make themselves known to the public: they neither advertise like their private counterparts do, nor do they attempt to lure in potential donors through institutional communication devices such as websites. It should also be remembered that communication differences are often also influenced by the regulations in force in the various countries, given that not all states allow public biobanks to use commercial infrastructures.

If the strength of a biobank is supposed to be in the quality and extensiveness of their sample collection, it appears that the public ones seem not to be interested in growth at all. Private biobanks are much more interested in the effectiveness of communication (via the web in particular) as they are commercial activities which must be supported economically. Public biobanks, on the contrary, being able to benefit from public livelihoods, can be more focused on research objectives rather than on the procurement of funds and increasing profits.

Another issue that creates differences between these two realities is represented by the different timing with which the results are communicated to customers/donors: DTC settings operate on a rapid turnaround time for their results, while public biobanks are aligned with the research funding cycle, which usually occurs every 3–5 years. This reflects the expectation of providing actionable results and may impact public trust towards these different structures.

All of the presented issues are exacerbated by an unwillingness to donate on the part of the general public, which has been shown to be higher among older, less-educated, minority-group and childless individuals, with a fear that this information will be used against them [10].

Currently, the entire business model of private biobanking is different from the public one, as the former are usually for profit entities and the latter are usually non-profit. Profits in the private sector come not only from the selling of DNA tests in a business-to-consumer approach, but also in a selling of data to other businesses, in a business-to-business approach. However, this two-sided model approach is growing in popularity faster than the rules and regulations are being put in place to control such a phenomenon [5]. Indeed, 23andMe’s database has become one of the largest in the world, with estimates of hundreds of thousands of samples. This set-up makes possible exchanges that would not otherwise have occurred, creating value for both sides and bringing the economics closer to efficiency.

Indeed, through indirectly connecting research teams and population data, it is able to create a value that otherwise would be lost because of the inability of public databanks to directly access the data they need to conduct their research for multiple reasons, including falsehoods which have been become widespread and are difficult to debunk, such as the case of the database project in Iceland or the case of the Sardinian DNA database [11,12].

In the public sector, instead, the biobanks only seem to represent an expense in the health institutions’ budget. A shift from a donor-only perspective to a customer-donor perspective would certainly turn the situation around, allowing the public sector to not only significantly increase the number of donations and the quality of their databases, but also to generate revenue to sustain themselves through the selling of these services, and the possibility to employ pre-existing infrastructures to provide the goods in question. A service of this kind by a public provider would also circumvent the issue of data selling, which is usually is found in private-sector databases and is not perceived positively by the public. Participants in a 2019 research study conducted by Milne et al. about trust in genomic data sharing were more prone to trust their doctor and less likely to trust other named entities, with company researchers the least likely to be trusted [13]. Certainly, this attitude on the part of the public would be a competitive advantage in a scenario with public biobanks more actively pursuing samples.

Moreover, when customers purchase a direct-to-consumer kit, they probably do not know that it will be used for research purposes and who will have access to their data, creating further ethical problems which are instead very important in the academic field and which have been at the center of a very heated debate [14].

Another very intriguing theme is that relating to the selection bias, as the clients donating to private biobanks are probably very different in baseline characteristics as compared to public databases. In this context, strategies should be sought to eliminate biases in the input elements of the creation and organization of a biobank, such as having the same type of basic information for all donors; asking donors for a minimum panel of biological matrices in order to have more uniform samples and thus avoid research waste; and improving the collaboration between public and private sectors, thus increasing the number of useful and suitable samples; implementing marketing and business approach studies on biobanks, both in the public and private sectors, to understand the different needs of donors and stakeholders and offer more targeted services, taking into account both the different needs of involved parties and the need to have good quality samples.

Private biobanks have the potential to become very important in the understanding of human genetics and its popularization, but, as underlined, their business will always be up for debate because of the services they offer. Private companies are an important factor in building large biobanks because of their innate ability to be in contact with their audience more than their public counterparts can. It is important to consider that the usefulness of private biobanks should be enhanced in regard to stimulating the tracing of a common governance framework, which can also implement the collaboration between the public and private sectors to benefit from the positive aspects of both realities [12]. When biobanks started to develop, ethical and legal attention was centered on donors’ involvement, and particularly on the abovementioned topics of transparency and public trust. Considering these topics to still be important despite the continuation of the debate and the broadening of horizons with respect to new regulations and new applications of biobanking, nowadays, we believe that, for private biobanking’s usefulness in public health, it is necessary to increase the opportunities for discussion between managers and stakeholders of biobanks for an integrated discussion on governance also from an ethical and regulatory point of view. This is reflected in how these DTC companies are faced with their research through their own biobank and how they manage the informed consent of customers so that they can maximize individual autonomy. These aspects are strongly linked to how these companies conduct their research with consumers’ data, regarding their status as research participants, which is reflected in transparency and trust. In fact, one of the main issues for researchers when reaching out or searching for suitable biobanks for their research purposes is the availability of clinical data and follow-up data. For almost all research questions a minimal set is necessary, while for most research questions, an even larger set of clinical data is absolutely necessary. The possibility at the very least should be offered to additionally collect these data, which often include, next to basic characteristics, variables such as disease outcomes; vital status; survival times; detailed diagnosis information or treatment information; or lifestyle factors such as smoking, alcohol use, physical activity, etc. For most public biobanks, a number of clinical characteristics may be available, or infrastructures to collect these data are in place. Some of these biobanks even work with dedicated questionnaires specifically aimed at the research questions of the public biobank. The topic of data availability for private biobanks is of paramount importance since these biobanks do not have access to the same infrastructure as public biobanks, do not have the same rights to retrieve clinical data, and are probably not allowed to retrieve a lot of clinical data upfront. With the harmonization of the rules, one could think of ad hoc questionnaires to be administered to all donors, both in the public and private sector, creating databases of information that can be used and shared on various platforms; in this context, the discussion could benefit from the extensive debate currently underway on the management and regulation of the use and management of health-related big data.

Next to the issue of obtaining clinical and follow-up data of sufficient quality for research purposes, another issue should be considered. When comparing biobanks, technical heterogeneity is a huge problem. Private biobanks, from their commercial point of view, often make different choices in regard to costs, obtaining as much DNA (or whatever is being collected in the biobank) as possible, technical possibilities, storage conditions, etc. It can be questioned whether results obtained from samples with a completely different technical work-up can be combined or compared. In addition, different research question could ask for a different technical work-up, something that is sometimes taken into account in public biobanks, evenly distributing samples over different work-ups for different questions. This, however, could be difficult to consider in private biobanks from their commercial point of view. Some authors have recently suggested borrowing some principles of business-focused practices in order to avoid the underutilization of biospecimens from bioresources and improve biobanks sustainability, for example, increasing business administration studies in the biobanks of public structures with different purposes and choosing the right model of supply of bio-resources among them in order to obtain high-quality samples that are useful for the intended research purposes [15,16].

The important role of the public sector lies in ensuring that adequate ethical standards are met regarding rights and social justice as perceived by the community. The way this can be achieved is through legislation which imposes limits on the liberty of action of private companies, and which maintains the market in a posture as competitive as possible, avoiding any kind of monopoly in order to balance conflicting rights, namely, the individual’s privacy and the prohibition of discrimination based on genetic information, and the community’s right to scientific progress in order to achieve better health treatment. The inclusion of public biobanks in the healthcare system represents an important step towards their financing and developing sustainability through stable funding, recognizing their importance in public health as systematic infrastructures. While for public biobanks the harmonization process has already been underway for some time, with a growing scientific and ethical debate, it is necessary that DTC companies using data repositories for research also adapt to harmonization policies. Harmonization between the two types of biobanks may undoubtedly increase the amount of usable data to implement the development of translational research, and may help to overcome some of the historical boundaries between private and public research. Indeed, the more competitive the market is, the more companies will pay attention in order to avoid scandals which would undermine their reputation, subsequently pushing them out of business. A defect in protection of donors’ data and the failure to respect participants’ autonomy would be fatal in this case and would have severe consequences on other companies as well, which would have to implement their measures to avoid such a risk.

On the other hand, the public sector could learn a lot from the success of their counterparts in the private sector. Through the adoption of better communication with the public, a less passive business approach focused on non-profit but profitable public biobanks and a proactive sharing of the results with the donors could cause an inversion of the current trend in the market that would favor the public sector. The biggest obstacle for the public sector, unsurprisingly enough, may be the public sector itself: excessive bureaucracy, lack of regulation of the private sector that allows for preying on unsuspecting consumers’ data, rigidity and resistance to change, and the lack of implementation of technologies that are already the norm everywhere else. Table 1 summarizes the main faults and strengths highlighted in this discussion.

## 4. Conclusions

The analysis of the differences between public biobanks and biobanks included in private DTC companies regarding transparency and public trust leads us to conclude that the long path of scientific and ethical debate on the organization and management of public biobanks can be a good background for reflection also in the private sector; the marketing and communication strategies of the private sector, although not perfect, can be borrowed from the public sector to improve citizen participation; a platform for dialogue, both technical–scientific and ethical, is indispensable between the public sector, the private sector and citizens to truly maximize both transparency and public trust in all contexts. A loyal cooperation of the public and private sectors in this field can develop into something extremely profitable and with very high social advantages. If the right equilibrium cannot be found, then the market will probably not be able to express all of its potentiality, and biobanking in general may have to deal with a lower public trust.

There is scarcity of information currently available to present a detailed and in-depth analysis on the comparisons between public and private (DTC) biobanks and of their ethical issues. Nevertheless, these information gaps could be filled by means of various strategies: large surveys involving different segments of the population with respect to their knowledge, use and expectations from both public and private biobanks; organization of surveillance programs, not only at national level but also at the European level, recognition of the numbers linked to biobanks (number of structures, samples donated, and samples used each year) and also, possibly of their profits; and the development of investment programs on the harmonization of the rules of the game between the public and private sectors, with the involvement of the stakeholders and not only of the directly interested parties and legislative bodies, to better understand both the expectations and the protection needs of the privacy policy determined by the public.

## Figures and Tables

**Table 1:** Faults and strengths of public and private biobanks in summary.

Private	Public
Business management strategies	Harmonization/regulation
Profit	No payment
Selection bias	Data availability
Image and communication strategies (testimonials/websites)	Information and consent
